# Analgesic and Anti-Inflammatory Effects of *Aucklandia lappa* Root Extracts on Acetic Acid-Induced Writhing in Mice and Monosodium Iodoacetate-Induced Osteoarthritis in Rats

**DOI:** 10.3390/plants10010042

**Published:** 2020-12-26

**Authors:** Hee-Geun Jo, Geon-Yeong Lee, Chae Yun Baek, Ho Sueb Song, Donghun Lee

**Affiliations:** 1 Chung-Yeon Central Institute, 64, Sangmujungang-ro, Seo-gu, Gwangju 61949, Korea; jho3366@hanmail.net; 2 Imsil County Health and Medical Center, Imsil 55927, Korea; kunyoung90@naver.com; 3 Department of Herbal Pharmacology, College of Korean Medicine, Gachon University, 1342 Seongnamdae-ro, Sujeong-gu, Seongnam 13120, Korea; cyning20@gachon.ac.kr

**Keywords:** osteoarthritis, *Aucklandia lappa*, analgesic, anti-inflammatory, MIA rats

## Abstract

Osteoarthritis (OA) is an age-related joint disease and one of the most common degenerative bone diseases among elderly people. The currently used therapeutic strategies relying on nonsteroidal anti-inflammatory drugs (NSAIDs) and steroids for OA are often associated with gastrointestinal, cardiovascular, and kidney disorders, despite being proven effective. *Aucklandia lappa* is a well-known traditional medicine. The root of *A. lappa* root has several bioactive compounds and has been in use as a natural remedy for bone diseases and other health conditions. We evaluated *the A. lappa* root extracts on OA progression as a natural therapeutic agent. *A. lappa* substantially reduced writhing numbers in mice induced with acetic acid. Monosodium iodoacetate (MIA) was injected into the rats through their knee joints of rats to induce experimental OA, which shows similar pathological characteristics to OA in human. *A. lappa* substantially reduced the MIA-induced weight-bearing of hind limb and reversed the cartilage erosion in MIA rats. IL-1β, a representative inflammatory mediator in OA, was also markedly decreased by *A. lappa* in the serum of MIA rats. In vitro, *A. lappa* lowered the secretion of NO and suppressed the IL-1β, COX-2, IL-6, and iNOS production in RAW264.7 macrophages activated with LPS. Based on its analgesic and anti-inflammatory effects, *A. lappa* could be a potential remedial agent against OA.

## 1. Introduction

Osteoarthritis (OA) is one of the long-term chronic degenerative bone joint diseases that affects the aged population over 65 [1]. Generally, OA patients are diagnosed with damaged cartilage, inflamed synovium, and eroded chondrocytes, which trigger pain and physical distress [2]. Arthritic pain is predominantly caused by the degeneration of cartilage in joints by inflammation, and when the cartilage is seriously damaged bones can collide with each other causing unbearable pain and physical hardship [3]. The involvement of inflammatory mediators with symptoms such as pain, swelling, and stiffness of the joint is well documented. In OA patients, inflammatory cytokines, which cause the erosion of cartilage and subchondral bone are found in the synovial fluid [4]. Two major complaints that OA patients generally have are pain and synovial inflammation. Therefore the primary goals of the current OA therapies are to lower pain and inflammation. [5]. Although the available OA treatments, including non-steroidal and steroidal drugs, have proven efficacies in alleviating pain and inflammation, the long-term uses of these drugs have severe health consequences such as cardiovascular, gastro-intestinal, and renal dysfunctions [6]. Thus, a more effective medicine with fewer side effects has to be developed for the treatment of osteoarthritis.

Natural health products are being increasingly popular for being safe and easily available [7]. Traditional Korean medicines have proven efficacies against several inflammatory diseases, including arthritis [8]. *Aucklandia lappa* DC. is known for its medicinal properties, such as enhancing the circulation of qi for relieving pain and soothing the stomach, and has been used traditionally as a natural analgesic [9]. Previous reports suggest that *A. lappa* possesses anti-inflammatory [10,11], analgesic [12], anticancer [13], and gastroprotective [14] effects. The various biological activities of *A. lappa* are caused by its major active compounds: costunolide, dehydrocostus lactone, dihydrocostunolide, costuslactone, α-costol, saussurea lactone and costuslactone [15]. Earlier studies claim that costunolide showed anti-inflammatory properties in lipopolysaccharide (LPS), which induced the macrophages through the regulation of NF-kB and heat shock protein pathway [16,17]. However, no study has investigated the potential activities of *A. lappa* for OA treatment. The present research has investigated the therapeutic effects of *A. lappa* against OA using (monosodium-iodoacetate) MIA and acetic acid-induced rodent models.

Monosodium-iodoacetate (MIA) is famously used to produce much of the pain behaviors and the pathophysiological features of OA in animals [18,19,20]. When injected into knee joints, MIA disarrays the chondrocyte metabolism and induces inflammation and inflammatory symptoms, such as cartilage and subchondral bone erosion, the cardinal symptoms of OA [18]. Writhing response induced with acetic acid is widely regarded as the simulation of peripheral pain in animals where the inflammatory pain can be quantitatively measured [19]. The mouse macrophage cell line, RAW264.7, is popularly used to study the cellular responses to inflammation. Upon activation with LPS, RAW264 macrophages activate inflammatory pathways and secrete several inflammatory intermediaries, as such TNF-α, COX-2, IL-1β, iNOS, and IL-6 [20]. This study has evaluated the anti-nociceptive and anti-inflammatory effects of *A. lappa* against OA in MIA animal model, acetic acid-induced animal model, and LPS-activated RAW264.7 cells.

## 2. Materials and Methods

### 2.1. Plant Material

The dried root of *A. lappa* DC. used in the experiment was procured from Epulip Pharmaceutical Co., Ltd., (Seoul, Korea). It was identified by Prof. Donghun Lee, Dept. of Herbal pharmacology, Col. of Korean Medicine, Gachon University, and the voucher specimen number was deposited as 18060301.

### 2.2. HPLC Analysis of A. lappa Extract

*A. lappa* was extracted using a reflux apparatus (distilled water, 3 h at 100 °C). The extracted solution was filtered and condensed using a low-pressure evaporator. *A. lappa* extract had a yield of 44.69% after freeze-drying under −80 °C. Chromatographic analysis of *A. lappa* was conducted with a HPLC connected using a 1260 InfinityⅡ HPLC-system (Agilent, Pal Alto, CA, USA). For chromatic separation, EclipseXDB C_18_ column (4.6 × 250 mm, 5 µm, Agilent) was used at 35 °C. A total of 100 mg of the specimen was diluted in 10 mL of 50% methanol and sonicated for 10 min. Samples were filtered with a syringe filter (Waters Corp., Milford, MA, USA) of 0.45 μm. The mobile phase composition was 0.1% phosphoric acid (A) and acetonitrile (B) and the column was eluted as follows: 0–60 min, 0%; 60–65 min, 100%; 65–67 min, 100%; 67–72 min, 0% solvent B with a flow rate of 1.0 mL/min. The effluent was observed at 210 nm using an injection volume of 10 μL. The analysis was performed in triplicate.

### 2.3. Animal Housing and Management

Male Sprague–Dawley (SD) rats aged 5 weeks and male ICR mice aged 6 weeks were purchased from Samtako Bio Korea (Gyeonggi-do, Korea). Animals were kept in a room using constant temperature (22 ± 2 °C) and humidity (55 ± 10%) and a light/dark cycle of 12/12 h. The animals were familiarized with the condition for more than a week before the experiment started. Animals had an ad libitum supply of feed and water. The current ethical rules for animal care and handling at Gachon University (GIACUC-R2019003) were strictly followed in all animal experimental procedures. The study was designed investigator-blinded and parallel trial. We followed the euthanasia method according to the guidelines of the Animal Experimental Ethics Committee.

### 2.4. MIA Injection and Treatment

Rats were randomly separated into 4 groups, namely sham, control, indomethacin, and *A. lappa*. Being anesthetized with 2% isofluorane O_2_ mixture, the rats were injected using 50 μL of MIA (40 mg/m; Sigma-Aldrich, St. Louis, MO, USA) intra-articularly into the knee joints to lead to experimental OA. The treatments were conducted as below: control and sham groups were maintained only with AIN-93G basic diet. Only, indomethacin group was provided with indomethacin (3 mg/kg) incorporated into AIN-93G diet and *A. lappa* 300 mg/kg group was assigned to AIN-93G diet supplemented with *A. lappa* (300 mg/kg). The treatments were continued for 24 days since the day of OA induction at the rate of 15–17 g per 190–210 g body weight on a daily basis.

### 2.5. Weight Bearing Measurement

After OA induction, weight-bearing capacity measurement of hind limbs of the rats was performed with the incapacitance-MeterTester600 (IITC Life Science, Woodland Hills, CA, USA) as scheduled. The weight distribution on hind limbs was calculated: weight bearing capacity (%)

$$=\frac{\mathrm{weight}\;\mathrm{on}\;\mathrm{right}\;\mathrm{hind}\;\mathrm{limb}}{\mathrm{weight}\;\mathrm{on}\;\mathrm{right}\;\mathrm{hind}\;\mathrm{limb}+\mathrm{weight}\;\mathrm{on}\;\mathrm{left}\;\mathrm{hind}\;\mathrm{limb}}\times 100$$

### 2.6. Serum Analysis

Blood was collected from the vein in abdominal area on 24 days after MIA injection and was left undisturbed for 30 min for clotting. Following centrifugation at 4000 rpm for 10 min, the serum was separated and stored at −70 °C. The level of pg was measured with a multiplex-assay kit (RnD Systems Inc. Minneapolis, MN, USA).

### 2.7. Micro-Computed Tomography Analysis

Micro-CT scan was performed to observe the structural changes in bone of the rats after the experiment. Right hind limbs of the rats were the severed, and the bones were fixed using formaldehyde (Sigma, USA). Micro-CT scanning was performed with a SKY scan 1176micro-CT system (Sky scan, Kartuizersweg, Belgium). 2D and 3D micro-CT images were analyzed with NR-econ (Sky scan v. 1.6.10.1), Data Viewer (Sky scan v.1.5.1.9), CTAn (Sky scan v.1.17.7.2), and CT-vol software (Sky scan v. 2.3.2.0). The imaging conditions were set as—X-ray source: 70 kV/355 µA; pixel size: 8.9 µm; filter: 0.5 mm aluminum; exposure time: 800 ms; rotation angle: 180° rotation angle using rotation steps of 0.4°.

### 2.8. Acetic Acid Induced Writhing Responses

ICR mice were grouped as control (water), ibuprofen (200 mg/kg; Sigma), and *A. lappa* (150, 300 and 600 mg/kg). Thirty min later, mice were given an intra-peritoneal injection of 0.7% acetic acid (10 mL/kg) and the writhing responses were measured after 10 min.

### 2.9. Cell Culture

RAW264.7 mouse macrophages used in the experiment were purchased from KCLB (Seoul, Korea). Macrophages were grown and incubated at 37 °C and 5% CO_2_ using DMEM medium added with 10% FBS and 100 IU/mL of penicillin-streptomycin (Gibco, Grand Island, NY, USA).

### 2.10. NO and Cytotoxicity Measurement

RAW264.7 cells (5 × 10^5^/well) were seeded into 6 well plate and cultured at 37 °C, 5% CO_2_ for 24 h. Incubated RAW264.7 cells were treated with 10 to 1000 µg/mL of *A. lappa* and 1 µg/mL of LPS and incubated for 24 h. Cell supernatants were transferred to a new well plate and mixed using Griess reagent (Sigma, USA) with 1:1 ratio, stored at room temperature for 10 min and the concentration of nitric oxide (NO) was measured at 540 nm. The cytotoxic effects of *A. lappa* were measured using the MTT assay at 540 nm. Following overnight seeding, 5 mg/mL of MTT reagent (Sigma, USA) was added to the cells and maintained at 37 °C, 5% CO_2_ for 1 h. 100ul of DMSO was added with the supernatant discarded mixture and kept for 10 min.

### 2.11. Quantitative Real-Time Polymerase Chain Reaction (qRT-PCR) Analysis

RAW264.7 cells were incubated with several concentrations of *A. lappa*, dexamethasone (1 µg/mL) as a positive control, and 1 µg/mL LPS for 24 h. Total RNA was extracted using QIAzol-Lysis buffer (Qiagen Ltd., Hilden, Germany). cDNA was synthesized with cDNA Reverse Transcription Kit (RnD System, USA). After amplifying the cDNA with Power-SYBRGreen Real Time-PCR Master mix (Applied Biosystem, Foster City, CA, USA) and primers (Bioneer, Daejon, Korea) quantitative real-time PCR (qRT-PCR) was conducted with Step One Plus qRT-PCR system (Applied Biosystem, USA). The PCR conditions were 95 °C for 15 s, and 60 °C for 60 s by 40 cycles after pre-incubating for 10 at 95 °C. The primer sequences are mentioned in Table 1.

### 2.12. Protein Expression Analysis

RAW264.7 cells were exposed to 30–300 µg/mL of *A. lappa* and LPS (1 µg/mL) for 24 h. After washing the cells with PBS, proteins were extracted with PRO-PREP^TM^ Protein Extraction Solution (iNtRON Biotech, Gyeoggi-do, Korea). Same amounts of proteins (µg) were loaded in polyacrylamide gel and moved to a nitrocellulose membrane. After blocking the membrane at room temperature for an hour with blocking solution, primary antibodies (iNOS, IL-1β and COX-2, Abcam, UK) were applied to react overnight at 4 °C. After adequate washing with TBST, membranes were probed with HRP-conjugated secondary antibody (Abcam, UK) in ambient temperature up to 2 h and then incubated with substrate-ECL Western blotting (Thermo Scientific Corp., Waltham, MA, USA). The Western blot image was analyzed with Amersham Imager^TM^ 600 (GE Healthcare Bio-sciences Corp., Seoul, Korea). Additionally, multiplex assay for IL-1β and IL-6 was carried out with Mouse Premixed Multi-Analyte Kit (RnD System, USA) in accordance with the manufacturer’s directions and the results were evaluated with a Luminex MAGPIX analyzer (Luminex Corp., Austin, TX, USA).

### 2.13. Statistical Analysis

For statistical analysis, Graph Pad Prism^®^ 5.0 (Graph Pad Software, CA, USA) was used. The significance difference was confirmed at *p* < 0.05 with Dunnett’s post hoc test and one-way ANOVA, all our data followed the normal distribution results and were expressed as mean ± standard error.

## 3. Results

### 3.1. HPLC Analysis

**Figure 1:**
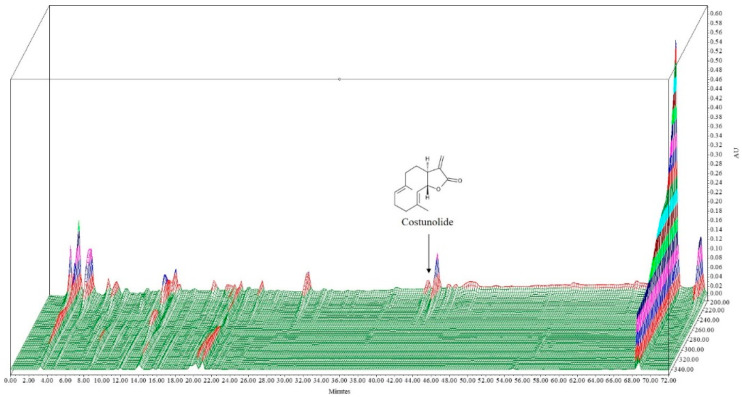
3-D HPLC chromatogram of *Aucklandia lappa* extract. X: retention time; Y: wavelength, and Z: absorbance unit.

In this study, costunolide was obtained and identified in *A. lappa* extract using HPLC-UV method. The extract contains 0.4 mg/g of costunolide. A 3D-HPLC chromatogram of the analysis along with the chemical structure of the constituent compound is presented in Figure 1.

### 3.2. Weight Bearing Distribution of MIA Rats

**Figure 2:**
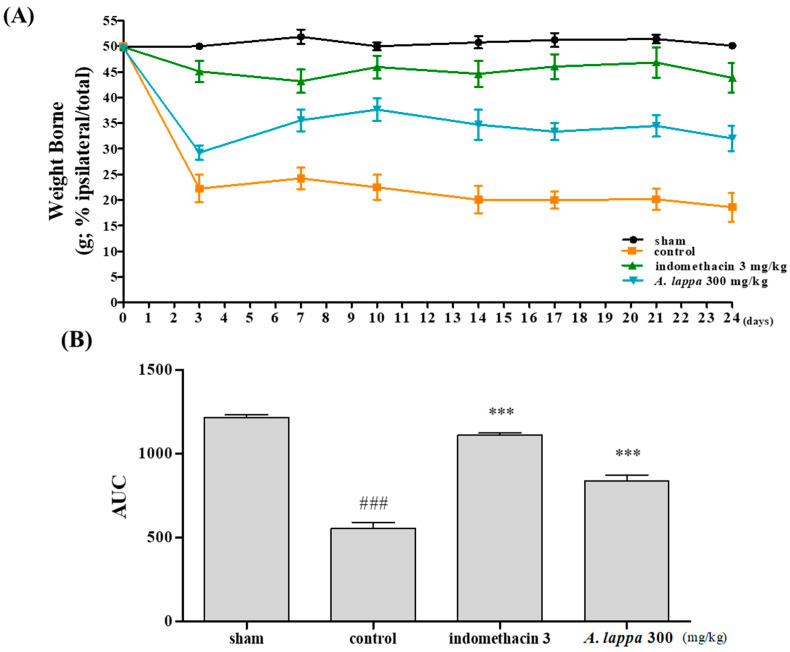
The effects of *Aucklandia lappa* on the hind paw weight-bearing capacities in monosodium-iodoacetate (MIA)-induced OA rats. (**A**) Weight-bearing ratios of the rats from 0–24 days with 3 mg/kg indomethacin or 300 mg/kg *Aucklandia lappa* and (**B**) AUC (area under the curve) measured with incapacitance meter tester. The significance was considered as ### *p* < 0.001 vs. sham, *** *p* < 0.001 vs. control.

Hind limb’s weight bearing is an index of join discomfort and arthritic pain and is commonly used in animal models to assess the analgesic properties of natural substances against OA. The distribution of the weight bearing ratio between the hind limbs was measured during 24 days after MIA injection. As illustrated in Figure 2A, MIA dramatically reduced the weight-bearing distribution in the control rats on day 3, and remained significantly lower thereafter as compared with the sham group. Noticeably, the *A. lappa*-treated rats showed a significant improvement in weight bearing. In particular, 300 mg/kg *A. lappa*-treated group had a similar level of recovery with the indomethacin 3 mg/kg-treated group (Figure 2B).

### 3.3. Prevention of Knee Joint Damage in MIA Rats

**Figure 3:**
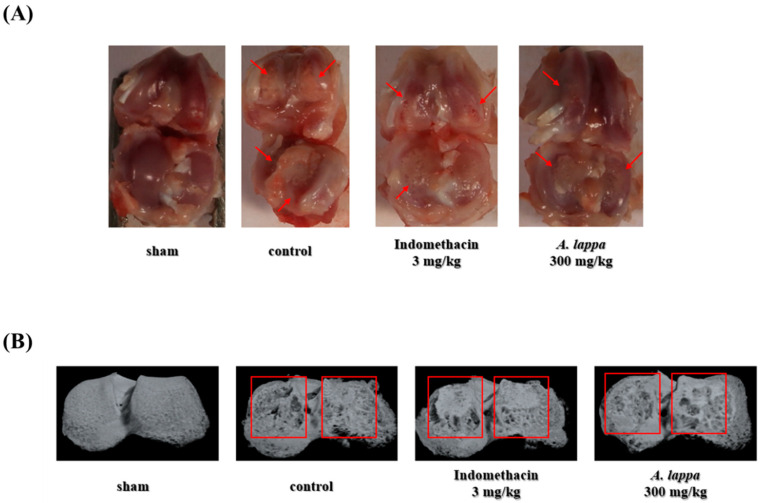
Observation of the structural properties of cartilaginous bone of Monosodium iodoacetate (MIA)-induced o OA rats. (**A**) Photograph of the right knee joints of the rats and (**B**) micro-CT images of the right tibia of the rats. Micro-CT scan was performed using SKY scan 1176 micro-CT system with these settings—X-ray source: 70 kV/355 µA; exposure time: 800 ms; pixel size: 8.9 µm; filter: 0.5 mm aluminum; pixel size: 8.9 µm; exposure time: 800 ms; rotation angle: 180° rotation angle with rotation steps of 0.4°.

The representative photographic images of the rat’s knee joints are shown in Figure 3. As illustrated in Figure 3A, the cartilage in the joint of sham rats was in a glossy, smooth state; whereas that of the control rat was clearly less polished along with visible damages in certain areas. The MIA-inflicted cartilage erosion was markedly recovered by *A. lappa* and indomethacin, and the recovery by *A. lappa* was higher than that of indomethacin. The micro-CT images show that a substantial loss of subchondral bone in the tibia occurred after MIA injection in the rats (Figure 3B), and the bone loss was significantly mitigated by *A. lappa*. Similarly, the cortical bone thinning in the MIA rats was markedly reversed with *A. lappa* supplementation.

### 3.4. Inflammatory Cytokines in MIA Rats

**Figure 4:**
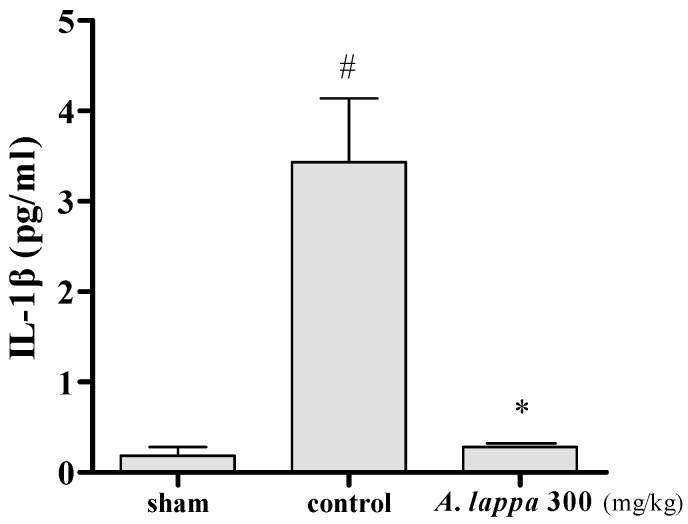
The levels of the inflammatory cytokine in the serum of monosodium-iodoacetate (MIA)-induced OA rats. Serum IL-1β levels were measured using Rat Premixed Multi-Analyte Kit and Luminex MAGPIX analyzer. # *p* < 0.05 vs. sham, * *p* < 0.05 vs. control.

This study has seen the inhibitory effect of *A. lappa* on the serum IL-1β level in the rats after OA induction. As shown in Figure 4, the serum IL-1β concentration of the control group was remarkably increased (*p* < 0.05) compared to the sham group. In contrast, *A. lappa* decreased IL-1β concentration in serum to the level of the sham group.

### 3.5. Acetic Acid Induced Writhing Responses

**Figure 5:**
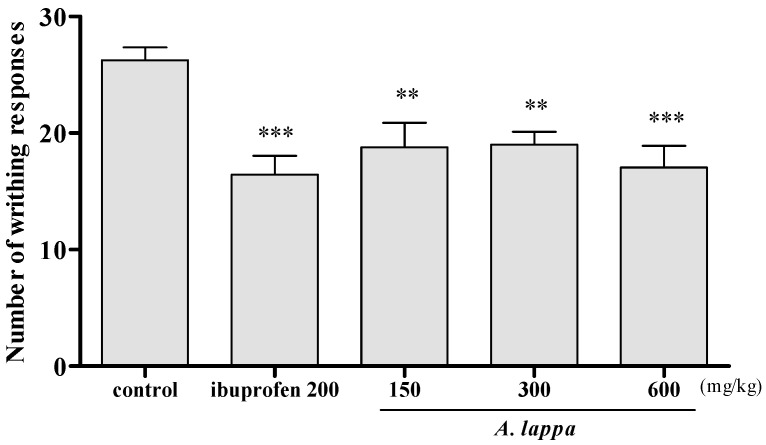
The acetic acid induced writhing responses in ICR mice. Mice were orally administered with ibuprofen and *A. lappa* and intra-peritoneally injected with 0.7% acetic acid. Each group had 7–8 mice; ** *p* < 0.05 vs. control, *** *p* < 0.001 vs. control.

Writhing responses in the acetic acid-induced mice were measured to investigate the analgesic effects of *A. lappa*. The average writhing responses in the control and the ibuprofen groups were 26.25 and 16.43, respectively. *A. lappa* administrated group showed a significant fall in the number of writhing in comparison with the control at all doses, especially, the 600 mg/kg *A. lappa* mouse showed the average writhing of 17.06, which was similar to the ibuprofen mouse (Figure 5). This result suggests that *A. lappa* has a pain-relieving effect on the acetic acid-induced writhing response.

### 3.6. Effects of A. lappa on Inflammatory Response in LPS-Activated RAW264.7 Cells

**Figure 6:**
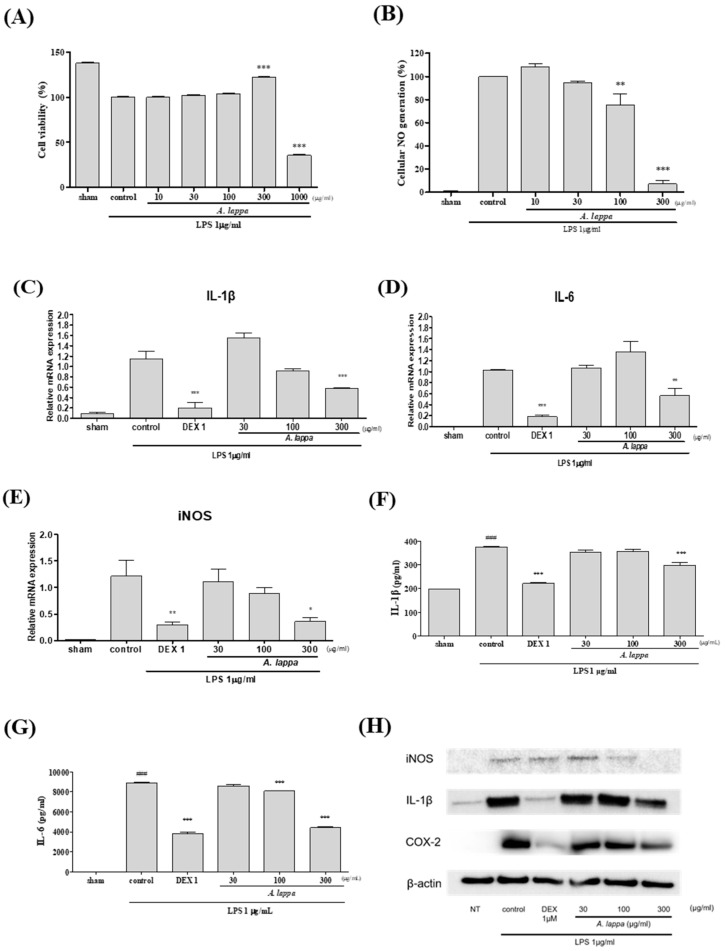
Effects of *Aucklandia lappa* treatment on (**A**) Cell viability (**B**) NO generation mRNA levels of (**C**) IL-1β, (**D**) IL-6, and (**E**) iNOS. Protein expression of (**F**) IL-1β, (**G**) IL-6, and (**H**) Western blot assay was performed for observing the protein expression. Protein expression of the cytokines was analyzed using multiplex analysis kit and through Western blotting. ### *p* < 0.001 vs. sham, * *p* < 0.05, ** *p* < 0.01, *** *p* < 0.001 vs. control.

In LPS-activated RAW264.7 cells, *A. lappa* showed anti-inflammatory actions by decreasing the NO and the inflammatory cytokine, as well as COX-2, iNOS, IL-1β, and IL-6. *A. lappa* did not express any cytotoxicity with up to 300 µg/mL (Figure 6A) in the cytotoxicity assay. The LPS-activated NO production was down-regulated by *A. lappa* in a dose-dependent way. Remarkably *A. lappa* at 300 µg/mL, reduced more than 80% of NO compared to the control (Figure 6B). *A. lappa* suppressed the mRNA expression of IL-1β, IL-6, and iNOS dose-dependently (Figure 6C–E). A significant decrease in the level of IL-6 by *A. lappa* was also noticed. To study the effects of *A. lappa* on the protein levels of pro-inflammatory cytokines, Western blot and multiplex analyses were performed. As shown in Figure 6F, G, *A. lappa* inhibited the increased production of IL-1β and IL-6 in LPS-activated RAW264.7 cells in a dose-dependent manner. Western blot images show that the iNOS, COX-2 and IL-1β were suppressed by *A. lappa* (Figure 6H).

## 4. Discussion

The present study has found that *A. lappa* increased weight-bearing and prevented the degradation of cartilage degradation and subchondral bone erosion in MIA rats. *A. lappa* also repressed the IL-1β production in MIA rats. Writhing responses in acetic acid-injected mice were decreased by *A. lappa*. In addition, *A. lappa* decreased the release of iNOS, COX-2 and IL-1β in LPS-activated RAW264.7 cells.

MIA is an alkylating agent that changes the thiol groups of proteins through S-carboxymethylation [21], and when administered to the joint cavity [22], it inhibits the glycolysis of cartilage cells and induces inflammation, which results in cartilage degradation and bone remodeling [23,24]. While several chemically and surgical induced OA models are available, the MIA-induced OA model is regarded as an archetypical system for studying osteoarthritis, as it can produce much of the symptomatic and pathophysiological features of human OA, including cartilage damage, inflammation, and subchondral bone degradation [25,26]. Reportedly, increase in weight-bearing in MIA rats represents the pain responses due to MIA injection [27,28]. Weight-bearing of the MIA rats in this study was remarkably increased by the administration of *A. lappa*, which was comparable to that of indomethacin. Therefore, the enhanced weight-bearing by *A. lappa* in the MIA rats suggests its analgesic effects against pain associated with OA.

Anti-inflammatory cytokines, namely IL-4,10, and 13 are released in the synovium, and are found in increased levels in OA patients [29,30]. These cytokines play significant roles in modulating inflammation by lowering the release of IL-1β, TNF-α, and MMPs and PGE_2_ and upregulating IL-1Rα and TIMP-1release [31,32]. Pro-inflammatory cytokines are known to play major roles in OA pathogenesis by inducing synovial inflammation, which leads to the cartilage destruction. IL-1β is one of the key mediators that enhance the OA inflammation [28]. IL-1β contributed to cartilage destruction by promoting protein breakdown enzymes and by suppressing the production of proteoglycan and collagen [29]. Our study observed that there was a dramatic increase of serum IL-1β in the MIA rats, and *A. lappa* administration caused a significant reduction of serum IL-1β. As the overproduction of IL-1β and other pro inflammatory cytokines promotes OA progression [30], the reduction of serum IL-1β by *A. lappa* implicates the increase in weight bearing of the rats by modulating the inflammatory reactions and the cartilage damage.

The damage and the structural modifications of cartilage and the adjacent bones in MIA rats are resonated with the histopathological characteristics of human OA. [31]. The losses of cartilage and subchondral bone are regarded as two of the prominent indicators of OA [32]. This study indicates that the pain relief by *A. lappa* was accompanied by a systemic improvement in the joint structure observed through the cross-sectional images and the micro CT examination of the knee joints of the rats. Degradation of cartilage is recovered in the *A. lappa*-treated MIA rats as compared with the non-treated MIA rats. Reportedly, MIA induces joint damage in animals similar to OA in humans by disrupting the proteoglycan matrix in the cartilage [33]. It has been suggested that the pathological conditions of MIA-injected rats induced mimic the disease conditions as well as the disease progression in the subchondral bone in human OA [34]. A marked improvement in the subchondral bone of the rats by *A. lappa* was also noticed in the micro CT analysis. The erosion of cartilage and the modification in subchondral structure in arthritic knees is generally resulted from the pro- inflammatory cytokines and mediators [35]. Moreover, an earlier study reported a decrease in cortical bone density as the osteoarthritis progressed in rats in comparison with the normal rats [36]. The authors have pointed out that the microarchitecture of subchondral bones can be modified by bone remodeling during OA pathogenesis. Similarly, our study also found that a cortical bone thinning occurred in the lateral and medial tibia in the MIA rats, which was significantly reversed by *A. lappa*.

Although central pain is clearly involved in OA, clinical studies indicate that peripheral factors are largely involved in causing OA pain [37]. Ethanolic extract of *A. lappa* has been reported to inhibit acetic-acid and hot-water-induced pain [38]. The analgesic effects of *A. lappa* against peripheral pain was evaluated in acetic acid-induced mice based on the writhing responses. It has been suggested that acetic acid induces the release of pain mediators, such as PGE_2_ and PGF_2α_ in the peritoneal cavity [39]. In this study, *A. lappa* dose-dependently decreased the number of writhing in the acetic acid-injected mice. Noticeably, *A. lappa* 600 mg/kg decreased the writhing similar to the level of the positive control. As suggested, acetic acid increases writhing responses in animals by releasing cytokines and pain mediators, which can quantitatively measure peripheral pain [40]. By decreasing the writhing responses in acetic acid-induced mice, *A. lappa* has shown remarkable analgesic effects against peripheral pain. In this study, the analgesic effects of *A. lappa* against peripheral pain might have involved in pain relief in MIA rats.

This study has found the anti-inflammatory effects of *A. lappa* in LPS-activated RAW264.7 cells. Us in vitro study showed that *A. lappa* inhibited the NO production and the protein and mRNA expression of iNOS, COX-2, IL-1β, and IL-6 in a dose-dependent manner. Costunolide, a representative sesquiterpene lactone of *A. lappa*, has been published to down-regulate the release of NO and TNF-α in LPS- activated macrophage [41]. Our in vitro study showed over-production of COX-2 and iNOS in joint rises pro-inflammatory cytokines and mediators in the synovium, which causes cartilage damage, and pain [42]. IL-1β, being the most crucial pro-inflammatory cytokine involved in OA progression enhances the release of COX-2, MMPs, IL-6, and iNOS [43]. This study has shown that *A. lappa* modulated the production of IL-1β and further pro-inflammatory cytokines and mediators.

The results of the current study indicated that *A. lappa* could reduce pain and prevented cartilage damage in MIA rats by prohibiting inflammatory reactions. Remarkably, the analgesic effects of *A. lappa* in MIA rats measured by weight-bearing was comparable to that of indomethacin. In addition, *A. lappa* substantially decreased the acetic acid-induced writhing in mice. The pain-relieving effects of *A. lappa* were coupled with the histological conservation of knee joints and the reduction of pro-inflammatory cytokines and mediators in the serum. Based on these results, it can be concluded that *A. lappa* could be a potential alternative therapy for OA inflammation and pain.

## Figures and Tables

**Table 1:** The primer sequences used for quantitative real-time RT-PCR.

IL-6	F	5′-ACCAGAGGAAATTTTCAATAGG-3′
	R	5′-TGATGCACTTGCAGAAAACA-3′
COX-2	F	5′-AACCGCATTGCCTCTGAAT-3′
	R	5′-CATGTTCCAGGAGGATGGAG-3′
TNF-α	F	5′-ATGGGCTTTCCGAATTCAC-3′
	R	5′-GAGGCAACCTGACCACTCTC-3′
IL-1β	F	5′-CCTAAAGTATGGGCTGGACTGT-3′
	R	5′-GACTAAGGAGTCCCCTGGAGAT-3′
iNOS	F	5′-CCCTTCCGAAGTTTCTGGCAGCAGC-3′
	R	5′-GGCTGTCAGAGCCTCGTGGCTTTGG-3′
GAPDH	F	5′-TGGCCTCCAAGGAGTAAGAAAC-3′
	R	5′-CAGCAACTGAGGGCCTCTCT-3′
